# Comparison of Cementless Calcar-Replacement Hemiarthroplasty With Proximal Femoral Nail for the Treatment of Unstable Intertrochanteric Fractures at Older Age Group

**DOI:** 10.7759/cureus.12854

**Published:** 2021-01-22

**Authors:** Anıl Agar, Adem Sahin, Orhan Gunes, Deniz Gulabi, Cemil Erturk

**Affiliations:** 1 Orthopaedics and Traumatology Department, Saglik Bilimleri University, Kanuni Sultan Suleyman Training and Research Hospital, Istanbul, TUR

**Keywords:** femur intertrochanteric fracture, calcar replacement, bipolar hemiarthroplasty, proximal femoral nail, harris hip score

## Abstract

Background: The aim of this study was to compare the outcomes of unstable intertrochanteric femur fractures treated with cementless calcar-replacement bipolar hemiarthroplasty (CRH) and proximal femoral nail (PFN) in elderly patients.

Methods: All consecutive unstable intertrochanteric fractures treated with cementless CRH or PFN at our institution between January 2015 and January 2019 were reviewed retrospectively. The primary outcome measures were postoperative complications, reoperation rate, and hip function. The secondary outcome measures were intraoperative blood loss, transfusion rate, surgical time, hospital stay, and two- year mortality.

Results: Ninety-four patients in the hemiarthroplasty group and 77 patients in the PFN group were included for analysis. There were no significant differences between the two groups regarding the complications, ASA score, and reoperation rate. Significant differences were found between hemiarthroplasty and PFN group in comparison of the average length of hospital stay (P < 0.05), time from hospitalization to operation (P < 0.05), intraoperative blood loss (P < 0.001), transfusion rate (P < 0.001), operation time (P < 0.001), Harris Hip Score (HHS; P < 0.001), and two-year mortality (P < 0.05).

Conclusion: Both hemiarthroplasty and PFN produce satisfactory results in surgically treated unstable intertrochanteric femur fractures in the elderly. Both groups are associated with their own complications, but in the PFN group, better functional results, less surgery-related trauma, and lower mortality rates are the main advantages.

## Introduction

The incidence of intertrochanteric femoral fractures, which is generally seen in the elderly population and causes significant mortality and morbidity in orthopedic practice, is increasing due to prolonged life expectancy [1]. As patients age and bone quality decreases, unstable pattern fractures occur. Intertrochanteric femoral fractures constitute approximately 45-50% of hip fractures in the elderly population [2] and 50-60% of these are of an unstable pattern [3]. The main goal of treatment of patients with this type of fracture is to return to their preoperative medical condition and daily activities [4]. Therefore, treatment aims for the patient to regain their pre-fracture level of activity by ensuring that the patient moves as soon as possible and to prevent the development of complications that may result in death due to immobility.

It is very important to provide effective and appropriate treatment in these fractures, which tend to be unstable due to decreased bone quality, especially due to the patient's age [5]. Many treatment methods such as proximal femoral nail (PFN), dynamic hip screw (DHS), external fixator, unipolar and bipolar hemiarthroplasty (BPH) have been used in the treatment of these fractures [6,7]. However, it is very difficult to provide stable fixation in these unstable class fractures due to decreased bone quality.

The purpose of treatment is to restore ambulation safely and efficiently while minimizing the risk of medical complications and technical failure. Patients can return to their pre-injury levels earlier by being treated with any of these methods, thereby eliminating postoperative complications caused by prolonged immobilization or implant failures [8].

The primary aim of this study was to compare the functional results of patients who underwent cementless calcar-replacement hemiarthroplasty (CRH) and patients who underwent PFN due to unstable intertrochanteric fractures over 65 years of age. The secondary objective was to compare intraoperative and postoperative complications in both groups.

## Materials and methods

A retrospective examination was made of patients who were hospitalized and treated surgically for intertrochanteric femur fractures at the University of Health Sciences, Kanuni Sultan Süleyman Training and Research Hospital between January 2015 and January 2019. The patients included were those over 65 years of age with an unstable intertrochanteric hip fracture (according to Evans classification; types 3, 4, and 5). Exclusion criteria were a history of osteoarthritis in the hip joint, pathological fractures, bilateral fractures, age <65 years, treatment with a method other than cementless CRH or PFN, fractures that developed secondary to a tumor, Paget's disease, or metabolic bone disease, multiple trauma with head and/or chest trauma, additional fractures of the same lower extremity, rheumatic disease, and inadequate follow-up.

The clinical and radiographic features of the patients on first admission, surgery, and final follow-up were evaluated retrospectively. The collected data of the patients included demographic characteristics, time from injury to surgery, length of stay in the hospital, type of fracture according to AO/ASIF and Evans classification, American Society of Anesthesiologists (ASA) physical condition classification (ASA grade), surgical procedure type uncemented CRH (TST, Istanbul, Turkey) or PFN with osteosynthesis (TST, Istanbul, Turkey), operation time, bleeding amount, blood transfusion, and complications.

All operations were performed by the same experienced surgical team. Before the surgery, the surgeon who performed the operation gave information to the patient about both surgical methods and it was decided as a result of a joint decision on which procedure to be performed. Low molecular weight heparin was started as antithrombotic prophylaxis from the time of hospitalization to all patients and was continued for one month. Antibiotic prophylaxis of cefazolin sodium was administered 30 minutes preoperatively and was continued for 24 hours postoperatively. Anteroposterior and lateral radiographs were obtained 24-72 hours postoperatively and were analyzed for reduction and position of the implant.

All patients were encouraged to do active and passive functional exercise from postoperative day 1 under the supervision of an experienced physiotherapist. Patients who underwent CRH were permitted full weight-bearing on the first postoperative day with the help of a walker. In the patient group applied with PFN, the patient was mobilized with partial weight-bearing on the postoperative first day with the help of a walker, and full weight-bearing was permitted at an average of 4 weeks according to the surgeon's decision in the follow-up examinations.

The patients were followed up every month for the first three months and then every three months for the first year and at six-month intervals thereafter. Complications were classified as problems not requiring revision surgery, such as bedsore, superficial infection, venous thromboembolism, and as problems requiring revision surgery, such as implant-related problems (cut-out, lateral sliding), deep infection, non-union, hip dislocation, or secondary fracture.

The functional outcomes of the patients were evaluated with the Harris Hip Score (HHS) at the final follow-up examination by a physician blinded to the surgical technique [9]. The average of the scores obtained was used for statistical analysis. The degree of pain, performance of daily activities, and range of motion were evaluated.

Statistical evaluation

Data obtained in the study were analyzed statistically using IBM SPSS Statistics 22.0 software (IBM Corp., Armonk, New York). Conformity of the data to normal distribution was evaluated with the Kolmogorov-Smirnov test. Descriptive statistics were stated as mean ± standard deviation (SD) values. The Student's t-test was used to compare quantitative data between two groups, and the Mann Whitney U test for two-group comparisons of non-normally distributed parameters. In the comparison of qualitative data, the Chi-Square test and the Continuity Correction (Yates) test were used. A value of p < 0.05 was accepted as statistically significant.

## Results

**Figure 1:**
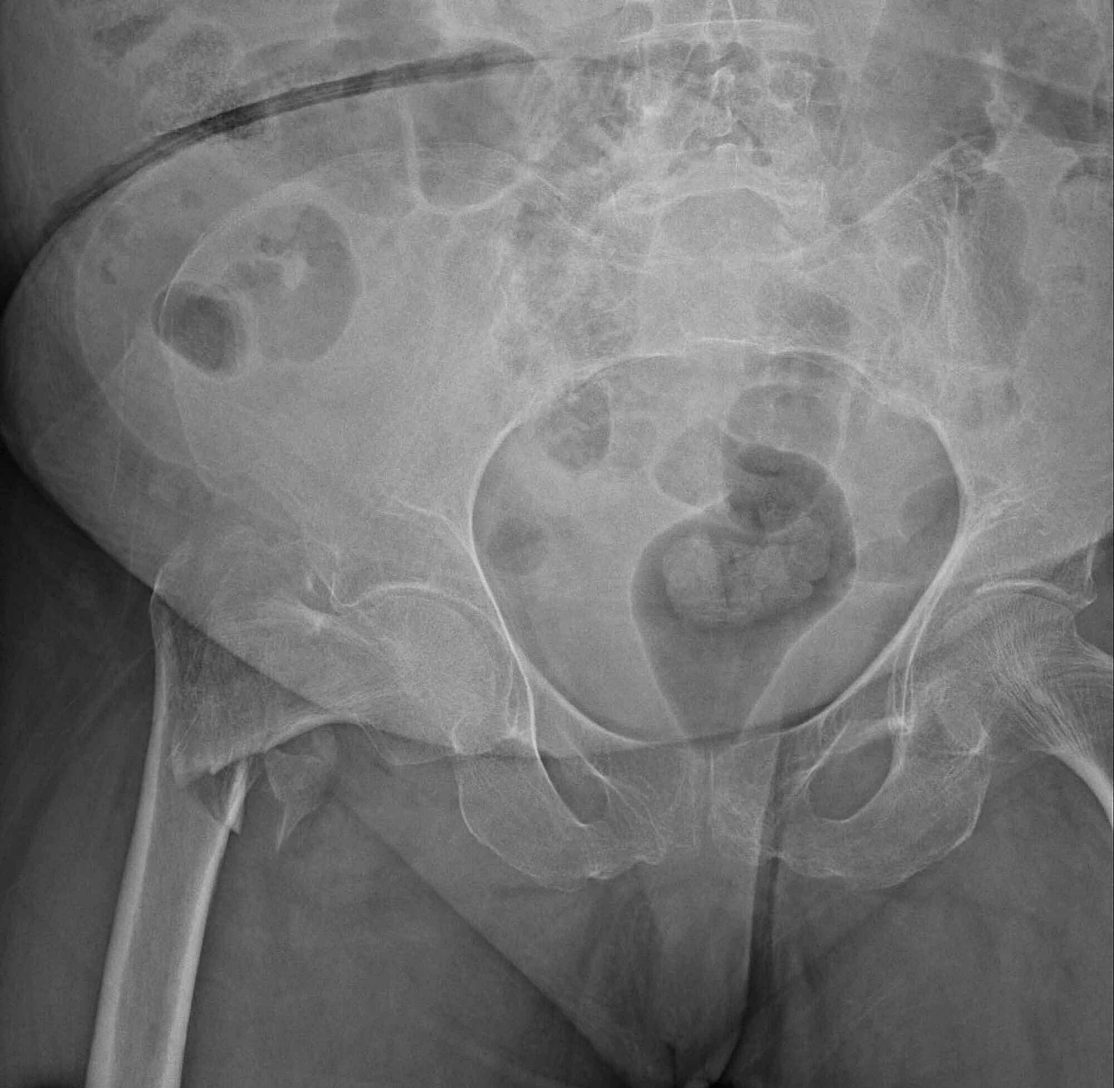
A 84-year-old female patient with a simple fall, right hip intertrochanteric fracture (type 5 according to Evans classification, type A2 according to AO classification). Cementless CRH applied to the patient. CRH: calcar-replacement hemiarthroplasty.

**Figure 2:**
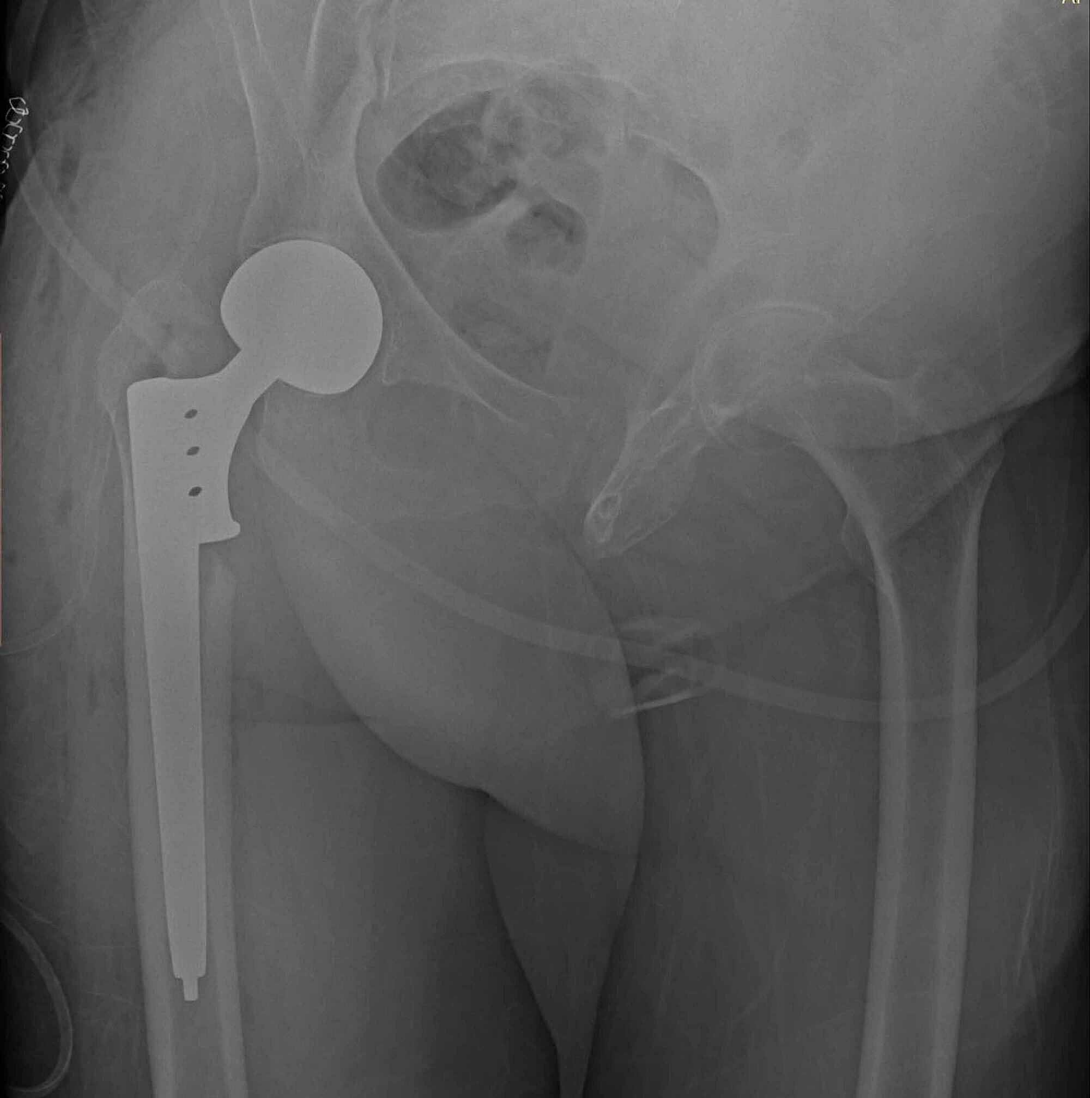
Postoperative first-day anterior-posterior hip radiography

**Figure 3:**
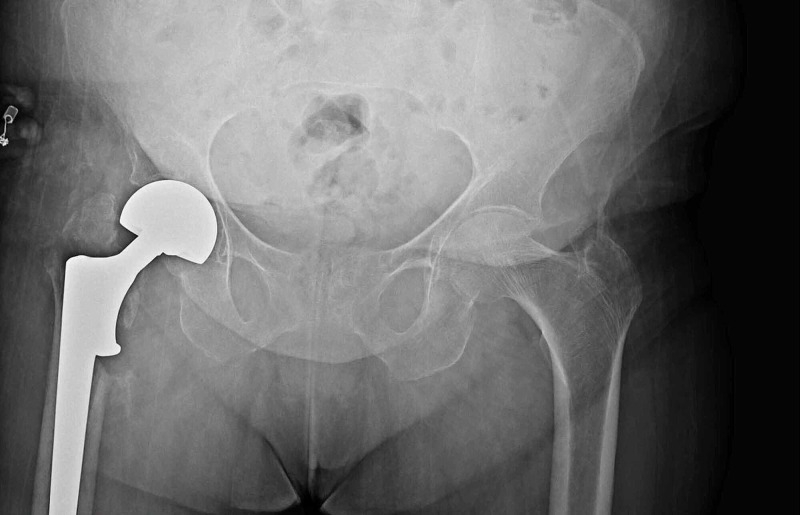
Anterior-posterior hip radiography at the third postoperative month of the patient

**Figure 4:**
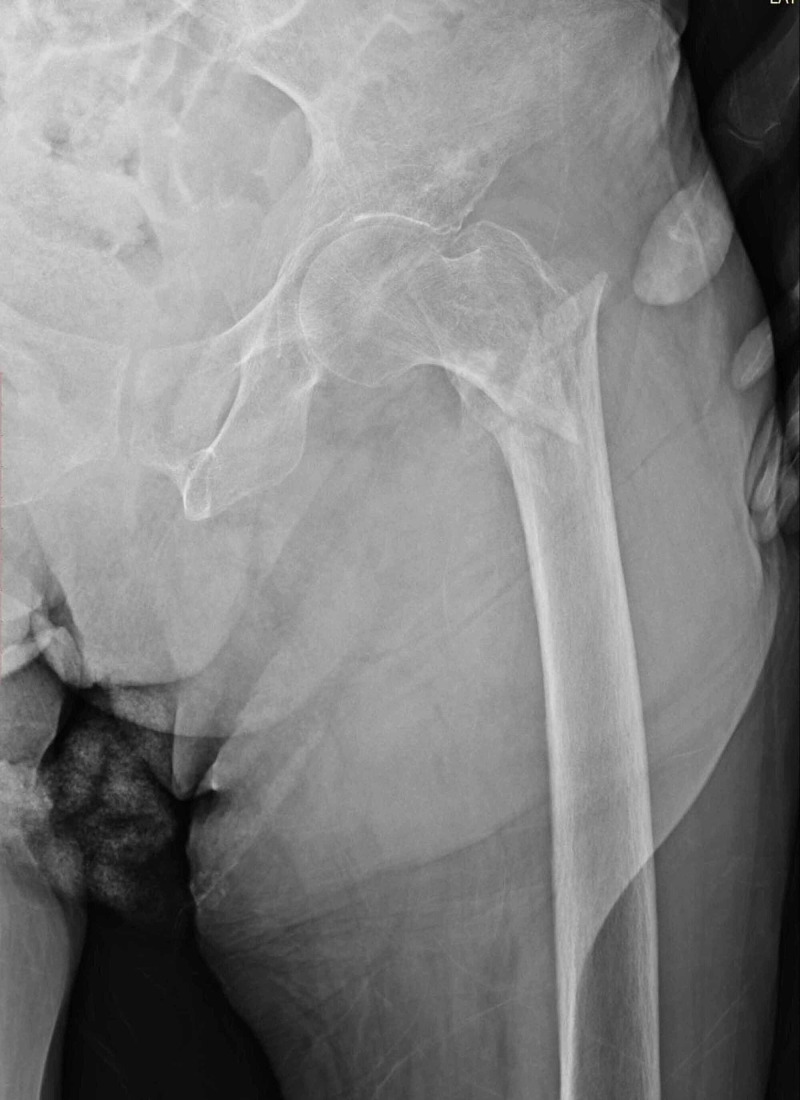
A 90-year-old female patient with a simple fall, left hip intertrochanteric fracture (type 4 according to Evans classification and type A2 according to AO classification). PFN applied to the patient. PNF: proximal femoral nailing.

**Figure 5:**
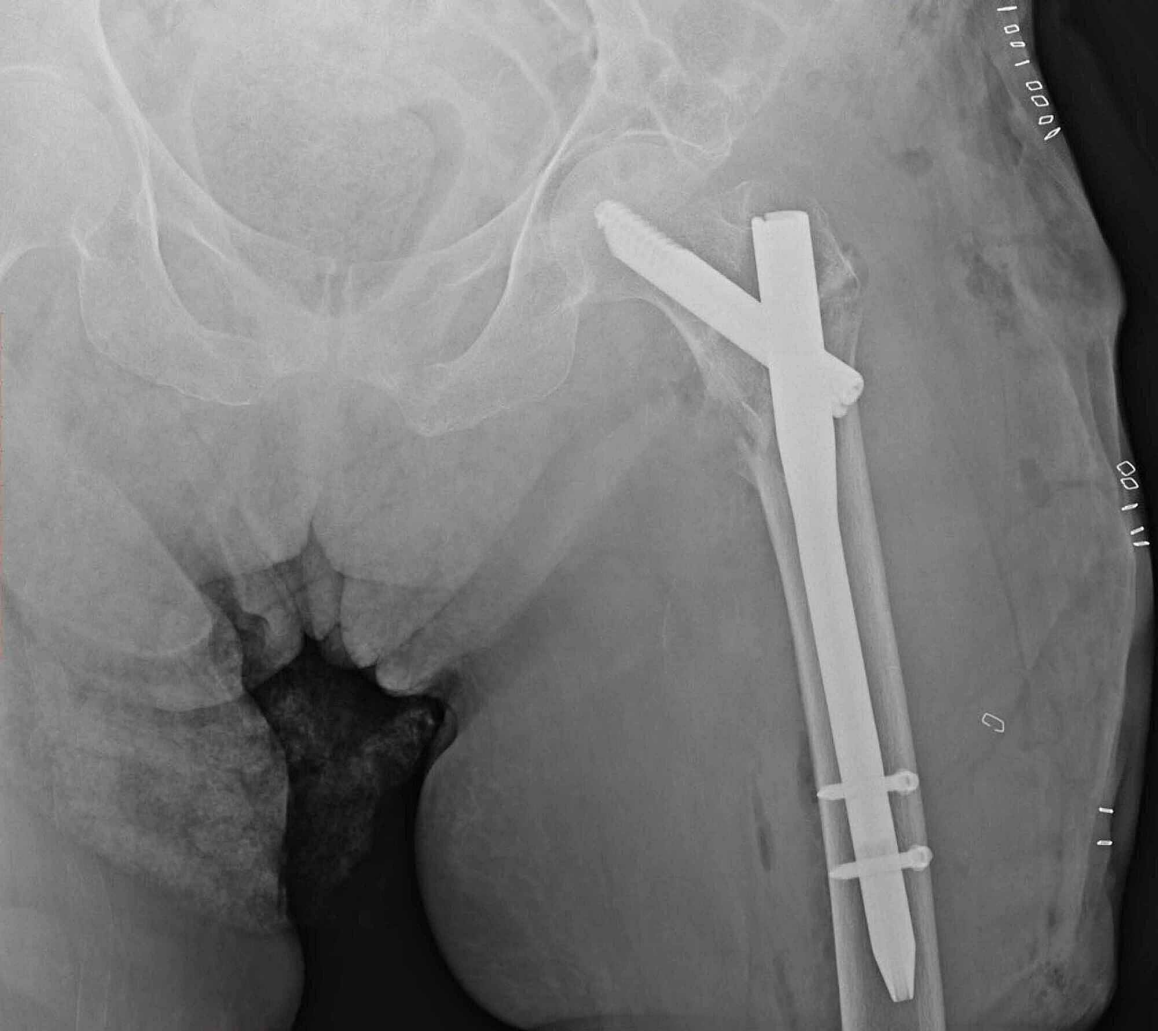
Postoperative first-day anterior-posterior hip radiography

**Figure 6:**
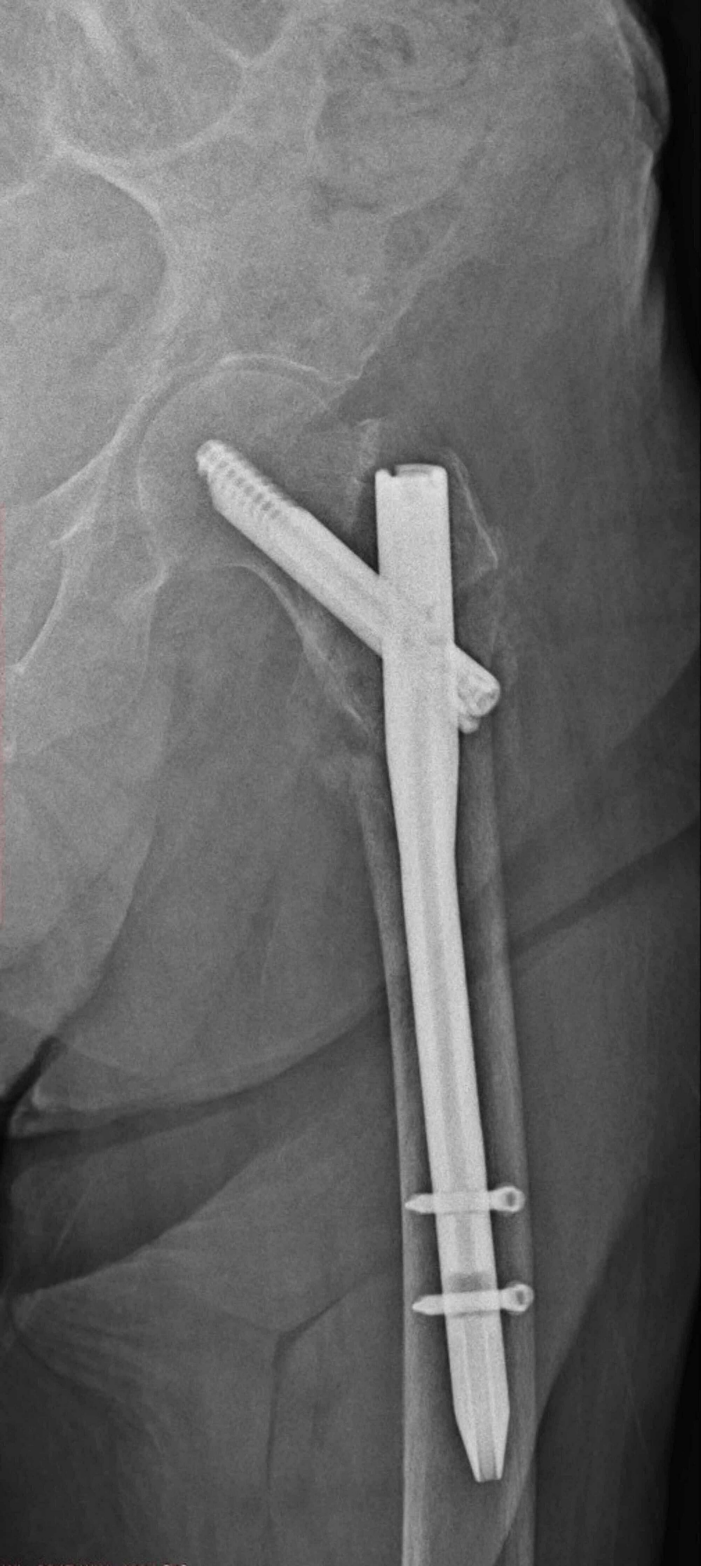
Anterior-posterior hip radiography at the third postoperative month of the patient

A total of 626 intertrochanteric fracture operations were performed between 2015 and 2019. The study included 171 patients who met the inclusion criteria, comprising 94 patients in the CRH group and 77 patients in the PFN group (typical cases are presented in Figures 1-6). The CRH group included 27 (28.7%) males and 67 (71.3%) females with a mean age of 82.2 years (range, 71-94 years). The PFN group included 21 (27.3%) males and 56 (72.6%) females with a mean age of 81.5 years (range, 65-99 years). The average follow-up time was 23.3 months (range, 15-35 months) in the CRH group and 21.9 months (range, 13-28 months) in the PFN group. There was no significant difference between the CRH and PFN group in terms of demographic data (Table 1).

**Table 1:** Evaluations by groups ^a^Student t-test; ^b^Mann-Whitney U test; ^c^Pearson chi-square test; dContinuity correction (Yates) test; *p<0.05; **p<0.01. CRH: calcar-replacement hemiarthroplasty, PFN: proximal femoral nail.

	Mean±SD (median)		p
	CRH	PFN	
Age	82.2±5.7	81.5±7.4	^a^0.504
The average length of hospital stay (day)	10.6±4.1 (10)	9.2±3.1 (9)	^b^0.009**
Time from hospitalization to operation (day)	6±2.4 (5)	5.9±2.9 (5)	^b^0.819
Intraoperative bleeding amount(ml)	514.6±63.7	127.3±42.6	^a^0.001**
Harris hip score	54.5±18.1	76.2±16.8	^a^0.001**
Operation time(min)	71.4±13.3	46.5±10.7	^a^0.001**
	n; %	n; %	
Gender			
Male	27; 28.7	21; 27.3	^c^0.834
Female	67; 71.3	56; 72.6	
Evans classification			
Type 3	27; 28.7	23; 29.9	^c^0.405
Type 4	52; 55.3	47; 61	
Type 5	15; 16	7; 9.1	
AO classification			
Type A2	79; 84	67; 87	^c^0.584
Type A3	15; 16	10; 13	
Pre-existing disease			
No	11; 11.7	5; 6.5	^d^0.368
Yes	83; 88.3	72; 93.5	
Blood transfusion			
No	30; 31.9	61; 79.2	^c^0.001**
1 unit	21; 22.3	14; 18.2	
2 unit	31; 33	2; 2.6	
≥3 unit	12; 12.8	0; 0	
ASA score			
2	15; 16	14; 18.2	^c^0.824
3	79; 84	63; 81.8	
Exitus			
No	55; 58.5	57; 74	^c^0.034*
Ex	39; 41.5	20; 26	
Revision			
No	86; 91.5	71; 92.2	^d^1.000
Yes	8; 8.5	6; 7.8	
Complication			
No	74; 78.7	63; 81.8	^c^0.614
Yes	20; 21.3	14; 18.2	

AO fracture classification was type A2 in 79 (84%) cases and A3 in 15 (16%) in the CRH group, and A2 in 67 (87%) cases, and A3 in 10 (13%) in the PFN group. According to the Evans classification, there were 27 (28.7%) type 3, 52 (55.3%) type 4, and 15 (16%) type 5 fractures in the CRH group. In the PFN group, 23 (29.9%) patients had type 3, 47 (61%) had type 4, and 7 (9.1%) had type 5 fractures. No significant difference was determined between the CRH and PFN groups according to fracture types.

ASA 2 status was determined in 17 patients and ASA 3 in 77 patients in the CRH group, and in the PFN group, 14 patients were ASA 2, and 63 were ASA 3. No significant difference was determined between the groups in respect of the ASA score (p > 0.05).

The average time from hospitalization to the operation was six days in the CRH group and 5.3 days in the PFN group. The average length of hospital stay was 10.6 days in the CRH group and 9.2 days in the PFN group (all patients were discharged from the hospital to their homes and did not continue rehabilitation in any hospital). The time from hospitalization to operation and the total length of hospital stay were statistically significantly longer in the CRH group than in the PFN group (p < 0.01)(Table 1).

In the CRH group, 83 patients had pre-existing comorbidities (68 patients had cardiovascular disease, 27 patients had diabetes mellitus, 19 patients had a respiratory disease, and 29 patients had the neurological disease), and 11 patients had no additional disease. In the PFN group, 72 patients had a comorbid disease (cardiovascular disease in 53 patients, diabetes mellitus in 24 patients, respiratory disease in 18 patients, and neurological disease in 24 patients), and 5 patients had no additional disease (Table 2). There was no statistically significant difference between the groups in terms of pre-existing comorbidities (p > 0.05).

**Table 2:** Pre-existing comorbidities CRH: calcar-replacement hemiarthroplasty, PFN: proximal femoral nail.

	n	
	CRH	PRN
Cardiovascular disease	68	53
Diabetes mellitus	27	24
Respiratory disease	19	18
Neurological disease	29	24
No additional disease	11	5

When the amount of intraoperative bleeding amount was evaluated (the amount of bleeding was determined by intraoperative gauze count and hemovac drain follow-up), it was determined as 514.6 ml in the CRH group and 127.3 ml in the PFN group. No blood transfusion was required in 30 (31.9%) patients in the CRH group, 1 unit was transfused in 21 (22.3%) patients, 2 units in 31 (33%) patients, and 3 or more units in 12 (12.8%) patients. In the PFN group, 61 (79.2%) of the patients did not require a blood transfusion, 1 unit was transfused in 14 (18.2%) patients, and 2 units in 2 (2.6%) patients. No patient in this group required 3 or more units of blood transfusion. When the amount of bleeding and blood transfusion rates of the patients were evaluated, the amount of blood loss in the CRH group was statistically significantly higher than in the PFN group (p < 0.01), and there was a statistically significant difference between the blood transfusion amounts (p < 0.01). The rate of no transfusion was higher in the PFN group than in the CRH group, and the rate of transfusion of 2 units or more was higher in the CRH group.

The mean operating time was 71.4 mins in the CRH group and 46.5 mins in the PFN group. The operating time of the CRH group was found to be statistically significantly longer than that of the PFN group (p <0.01).

Postoperative complications were determined in 20 (21.3%) patients in the CRH group (six superficial infections, three deep infections, eight thromboembolic complications, five bedsores), and in 14 (18.2%) patients in the PFN group (five superficial infections, five thromboembolic complications, four bedsores). No statistically significant difference was determined between the groups in respect of the incidence of postoperative complications (p > 0.05).

When the secondary operation-revision rates of the patients were evaluated, revision surgery was performed in eight (8.5%) patients in the CRH group (four deep infections, two hip dislocations, two periprosthetic fracture) and in six (7.8%) patients in the PFN group (six cut-outs). There was no statistically significant difference between the revision status according to the groups (p > 0.05).

The two-year survival was evaluated and mortality was seen in 39 (41.5%) patients in the CRH group and 20 (26%) patients in the PFN group. The incidence of exitus was significantly higher in the CRH group than in the PFN group (p <0.05).

In the evaluation of HHS at the final follow-up examination of the patients, the mean HHS was 54.5 in the CRH group and 76.2 in the PFN group. The HHS of the PFN group was statistically significantly higher than that of the CRH group (p < 0.01).

## Discussion

The main findings of the present study are as follows: less total length of hospital stay, better functional outcomes, less surgery-related trauma, and lower mortality rates in the PFN group.

Intertrochanteric femoral fractures account for 3.6% of all extremity fractures and 45-50% of all hip fractures in the elderly population [10]. It is very difficult for these elderly patients to return to the pre-injury physical condition due to frequent osteoporosis and delayed fracture healing, which can result in complications and high mortality rates [11]. Treatment of intertrochanteric fractures is surgery, and objective and careful preoperative evaluation of the fracture is required to develop an appropriate treatment plan [12]. Common intertrochanteric fracture treatments include intramedullary fixation (Gamma nail, PFNA), plate fixation (DHS, dynamic condylar screw [DCS]), and BPH. Many elderly patients also have osteoporosis, so fixation may not always be as effective as wished. Most authors currently recommend hemiarthroplasty and PFN as the first surgical option for the treatment of elderly patients with an unstable intertrochanteric fracture [13,14]. As a minimally invasive surgery, PFN has good biomechanical properties, making it a preferred method for unstable intertrochanteric fractures associated with osteoporosis [15]. To allow earlier postoperative weight-bearing and to avoid excessive collapse at the fracture site, some surgeons have recommended prosthetic replacement, especially with a calcar-replacement or head and neck-replacement type of prosthesis, for the treatment of unstable intertrochanteric fractures [1,16].

In the present study, similar to the literature, there was no significant difference between the patient classifications of fractures and ASA scores [17,18]. Since the focus in this study was on the results of patients over 65 years of age, these patients are more likely to have an additional disease, and intertrochanteric femoral fractures tend to be unstable. In a retrospective study by Zhou et al. [17], there was determined to be no significant difference between hemiarthroplasty and PFN groups with respect to ASA scores and fracture types. Gormeli et al. also found no difference between ASA scores and fracture types in a retrospective study [18].

In the current study, the average length of hospital stay was 10.6 days in the CRH group, while it was 9.2 days in the PFN group. It has been stated in the literature that the duration of hospitalization of extracapsular hip fractures is similar for both hemiarthroplasty and internal fixation groups [17]. In the present study, the duration of hospitalization in the hemiarthroplasty group was found to be significantly longer than that of the PFN group, and the time from hospitalization to surgery was also significantly longer in the hemiarthroplasty group. The longer hospital stay of the CRH group can be attributed to the higher rate of blood transfusion required for patients in this group and also shorter total hospitalization time could be related to pre-operative hospitalization time.

Since intertrochanteric femoral fractures usually appear at older ages when the bone structure is weakened, the percentage of pre-existing comorbidities in these patients is high. In a study by Jolly et al. [19], it was reported that 64% of patients in the hemiarthroplasty group and 60% of patients with PFN had an additional disease. In a retrospective study by Kezmezacar et al. [20], it was similarly stated that there was no statistically significant difference between the patients' comorbidities. In the current study, pre-existing comorbidities were found in 88.3% of the patients in the CRH group and in 93.5% of the patients in the PFN group, with no significant difference determined between the groups.

In a randomized prospective study, Kim et al. [21] stated that the duration of surgery, the amount of bleeding, and the amount of blood transfusion were significantly higher in the hemiarthroplasty group than in the PFN group. There was also determined to be no significant difference between the groups in complications seen in the postoperative period. In the present study, the duration of surgery, the amount of bleeding, and the amount of blood transfusion were significantly higher in the hemiarthroplasty group, and there was no significant difference between the groups in respect of complications seen in the postoperative period.

In similar studies in the literature, operation times have been found to be longer in patients with hemiarthroplasty than in the group with PFN [19,21]. In the current study, similar to the literature, the operating time of patients who underwent hemiarthroplasty was found to be significantly longer compared to patients who underwent PFN.

Following intertrochanteric femoral fractures, early complications may occur that do not require a secondary operation such as superficial infection, venous thromboembolism, and bedsores. In their prospective study, Kim et al. [21] encountered early complications in 12 of 29 patients who underwent hemiarthroplasty and in 8 of 29 patients who underwent PFN. Lou et al. [22] also stated in their retrospective study that there was no statistically significant difference between the rates of complications. In the present study, early complications were observed in 21.3% of patients in the hemiarthroplasty group and in 14% of patients with PFN, with no significant difference determined between the groups.

Intertrochanteric femur fractures are difficult to reduce intraoperatively and screw loosening and cut out in femoral necks can be often seen with severe osteoporosis. Studies have shown that the use of PFNs in the treatment of intertrochanteric fractures has a failure rate of 7.1-12.5% [23,24]. In contrast, hemiarthroplasty can quickly restore hip function, so it is mainly used to treat femoral neck fractures in the elderly, including unstable intertrochanteric fractures and failure of intertrochanteric fracture fixation [1]. In the current study, eight (8.5%) patients in the CRH group and six (7.8%) patients in the PFN group developed complications requiring revision and there was no statistically significant difference between the two groups.

Kesmezacar et al. [20] compared internal fixation and hemiarthroplasty in elderly patients with pertrochanteric femur fractures and reported that a mortality rate of 48.8% in the first six months in patients undergoing endoprosthesis, while this rate was 34.2% in patients with internal fixation. Kim et al. [21] compared the results of hemiarthroplasty and PFN with internal fixation in unstable pertrochanteric fractures and reported mortality as 55% in the arthroplasty group in the third year, but 17% in the PFN group. In the present study, the two-year mortality rate was significantly higher in the hemiarthroplasty group than in the PFN group.

In a retrospective study by Gormeli et al. [18], the HHS was found to be higher in the PFN group but there was no significant difference compared to the hemiarthroplasty group. In a prospective study by Jolly et al. [19] comparing hemiarthroplasty and PFN, the HHS at the end of the first postoperative year was better in the PFN group. Similarly, in the present study, the HHS at the end of the first year was found to be significantly higher in the PFN group than in the hemiarthroplasty group.

This study had some limitations, primarily the retrospective design, small patient group, and mid-term follow-up. Long-term analyses are unlikely in an elderly patient population due to the short life expectancy.

## Conclusions

In conclusion, both hemiarthroplasty and PFN produce satisfactory results in surgically treated intertrochanteric femur fractures. Both groups are associated with their own complications, but in the PFN group, better functional results, less surgery-related trauma, and lower mortality rates are the main advantages. Therefore, the clinician should select the ideal method for each patient, but it can be considered that internal fixation may be a more effective, and appropriate treatment method for elderly patients with an extracapsular proximal femur fracture.
